# Quality assurance target for community-based breast cancer screening in China: a model simulation

**DOI:** 10.1186/s12885-018-4168-1

**Published:** 2018-03-07

**Authors:** Lan Yang, Jing Wang, Juan Cheng, Yuan Wang, Wenli Lu

**Affiliations:** 1 Department of Epidemiology and Health Statistics, School of Public Health, Tianjin Medical University, 22 Qixiangtai Road, Heping District, Tianjin, 300070 People’s Republic of China; 2 Tianjin Binhai New Area Tanggu Center for Disease Control and Prevention, Tianjin, 300451 China; 3 Collaborative Innovation Center of Chronic disease prevention and control, Tianjin Medical University, Tianjin, 300070 China

**Keywords:** Breast cancer, Screening, Cost-effective

## Abstract

**Background:**

We aimed to clarify the feasibility of a community-based screening strategy for breast cancer in Tianjin, China; to identify the factors that most significantly influenced its feasibility; and to identify the reference range for quality control.

**Methods:**

A state-transition Markov model simulated a hypothetical cohort of 100,000 healthy women, the start aged was set at 35 years and the time horizon was set to 50 years. The primary outcome for the model was the incremental cost-utility ratio (ICUR), defined as the program’s cost per quality-adjusted life year (QALY) gained. Three screening strategies providing by community health service for women aged 35 to 69 years was compared regarding to different intervals.

**Result:**

The probability of the ICUR being below 20 272USD (i.e., triple the annual gross domestic product [3 GDPs]) per QALY saved was 100% for annual screening strategy and screening every three years. Only when the attendance rate was > 50%, the probability for annual screening would be cost effective > 95%. The probability for the annual screening strategy being cost effective could reach to 95% for a willingness-to-pay (WTP) of 2 GDPs when the compliance rate for transfer was > 80%. When 10% stage I tumors were detected by screening, the probability of the annual screening strategy being cost effective would be up to 95% for a WTP > 3 GDPs.

**Conclusion:**

Annual community-based breast cancer screening was cost effective for a WTP of 3 GDP based on the incidence of breast cancer in Tianjin, China. Measures are needed to ensure performance indicators to a desirable level for the cost-effectiveness of breast cancer screening.

**Electronic supplementary material:**

The online version of this article (10.1186/s12885-018-4168-1) contains supplementary material, which is available to authorized users.

## Background

Breast cancer is now the most common cancer in Chinese women, with cases accounting for 12.2% of all newly diagnosed breast cancers and 9.6% of all deaths from breast cancer worldwide [1]. There is solid evidence supporting the value of diagnosing cancer early, and Western societies have produced guidelines on early detection [2, 3]. Indeed, breast cancer screening by population-based mammography (MAM) has been proven to reduce mortality in several randomized trials in developed Western countries [4, 5]. However, in developing countries, a screening strategy that combines clinical breast examination (CBE) and breast ultrasonography (USG) may be a more acceptable approach [6, 7].

There is no nationwide screening program for breast cancer in China at present [1], although population-based studies of CBE combined with diagnostic USG, MAM, or both are currently in progress [1, 8]. Local governments have also sponsored community-based breast cancer screening programs in several urban cities despite doubts about the efficacy of CBE for early detection in diverse Chinese populations. Interestingly, a population-based study of these breast cancer screening programs provided good performance results, with a sensitivity and specificity of 70% and 90%, respectively [9]. Nevertheless, breast cancer screening, especially when using CBE, may not be as effective in clinical settings as it is in trial settings [10].

In Tianjin, the fourth largest city in China in terms of urban population, initial breast cancer screening is provided at community health services without the support of MAM or USG services from 2009.CBEs are done by trained health care providers funded by the local government as part of the basic public health service package. Women determined by their primary physicians to have a lesion that is suspicious or highly suggestive of a malignancy are then referred for further diagnostic tests and treatment, but these are not part of the basic public health service package and must be paid by patients or their medical insurance.

The government and public have high expectations of community health services to protect women’s health by detecting breast cancer early. However, community health services may not always be able to fulfill this role because of their limited diagnostic capacity and the shortage of doctors compared with the considerable number of women eligible for screening [11]. This is compounded by the fact that there has been no report on the feasibility of the current breast cancer screening program.

The primary aim of a breast screening program should be to reduce mortality from breast cancer through early detection. Unfortunately, it would take decades to confirm the effectiveness of such a screening program based on mortality indicators alone. By contrast, quality assurance allows the use of alternative performance indicators for quality control and evaluation [12–14]. There is a growing need to develop approaches that reflect the relationships between performance indicators and the feasibility of a screening strategy. Such an approach should help determine those factors that should be considered most important in practice, and should help set reasonable goals for the relevant indicators.

A decision-analytic model was used in the current study to predict the feasibility of a community-based breast cancer screening strategy in China. In addition, a sensitivity analysis approach was used to identify the relevant factors that significantly influenced the feasibility of such screening in community health services and to identify the optimum control ranges of those factors.

## Methods

### Community-based screening strategy

**Fig. 1:**
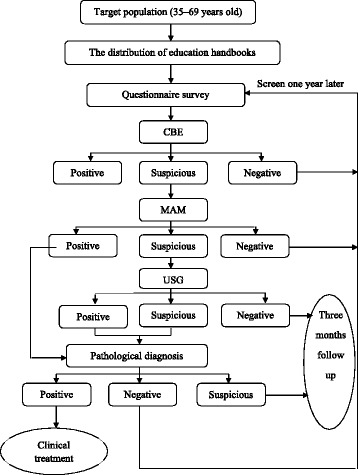
The Screening Flow Chart (Conventional Version)

In Tianjin, China, a community-based breast cancer screening program for women aged 35–69 years has been conducted for a seven-year period using CBE (Fig. 1). No inter-screening interval period has been clearly defined. When women have a positive CBE result, they are advised to undergo diagnostic USG or MAM.

### Markov decision tree

A state-transition Markov model was developed that consisted of two linked modules: (a) a breast cancer progression model, and (b) a screening model. The breast cancer progression model consisted of eight main health states: well; ductal carcinoma in situ (DCIS); the four invasive cancer stages defined by the American Joint Committee on Cancer (stage I − IV); and two death states, including death from breast cancer and death from causes unrelated to breast cancer. Women who were not detected as having breast cancer could present with signs and symptoms of breast cancer and could progress through each cycle. It was assumed that breast cancer deaths could occur only among women with stage IV disease, except when death was from natural causes. The transition probabilities from health status to breast cancer was calculated with age specified incidence and afterwards stage distribution in order to assign the stage in which cancer was detected (by screening or symptoms). Age-specific death rate from all causes and the age-specific death rate from breast cancer were used to estimate the transition probabilities from health or breast cancer to death (Table 1). The probabilities of stage progression were taken from a previous study in Chinese women [15]. (b) The screening model was based on whether in a screening year, women went into the model and accepted CBE, and whether women with positive or suspicious CBE results underwent MAM. Women with suspicious MAM results also underwent a supplementary USG examination. Those with positive results on MAM and USG would either receive breast biopsy or undergo prompt surgery. Women who had positive MAM results but negative USG results, and women who had suspicious biopsy results would receive an extra follow up USG after three months. Women who were not detected as having breast cancer could present with signs and symptoms of breast cancer and could progress through each cycle (clinical detected).

**Table 1:** Clinical and cost parameter estimates for the base case and sensitivity analyses

Parameter				Ref.
The distribution of invasive Breast Cancer stages	Screen	No screen	Distribution	[43, 44]
Stage I	0.360	0.203	Dirichlet [15]	
Stage II	0.490	0.541		
Stage III	0.144	0.237		
Stage IV	0.006	0.019		
Screen method test characteristics	Sensitivity	Specificity		
CBE	0.431 (0.335–0.528)	0.994 (0.994–0.995)	Uniform [15]	[18]
A series of CBE, MAM and ultrasound connection	0.330 (0.238–0.422)	0.999 (0.999–1.000)		
A series of CBE and MAM connection	0.360 (0.256–0.454)	0.999 (0.999–1.000)		
Stage progression transition probabilities				[15]
Stage I-IV	0.01		Invariant [15]	
Stage II-IV	0.08			
Stage III-IV	0.21			
Compliance rate	0.5–1		Uniform [15]	[18]
Attend rate	0.3–1		Uniform [15]	
Transition probabilities of breast cancer(Rate per 100,000 women)	All-cause mortality		Breast cancer mortality	
35-	53.86		3.78	[17]
40-	95.25		6.90	
45-	149.34		12.66	
50-	212.43		16.57	
55-	348.31		22.74	
60-	604.84		23.49	
65-	1030.55		23.95	
70-	2036.08		25.86	
75-	3783.51		31.57	
80-	6997.94		40.36	
> 85	13,602.90		48.85	
Cost components^a^	Cost			
Management cost and cost for CBE	$4.3			[20]
Cost of evaluating abnormal CBE				
MAM	$29.0			[20]
USG	$10.2			
Cost of biopsy	$174.3			
Treatment and follow-up	Treatment cost		Follow-up cost	
DCIS	$1607.2		$1712.7	[21, 22]
Stage I	$1940.7		$4022.8	
Stage II	$1960.1		$5653.7	
Stage III	$1902.9		$6481.7	
Stage IV	$1566.7		$4584.5	

Abbreviations: *CBE*; Clinical breast examination, *MAM*; Mammography, *USG*; Ultrasonography, *DCIS*; Ductal carcinoma in situ

^a^Exchange rate 6.8858 RMB = 1 US Dollar

During the simulation, one-, two-, and three-year intervals were compared because the inter-screening interval was not clearly defined in clinical practice. The model tracked a cohort of 100,000 women, without breast cancer, aged 35 years. The timeframes for exposure to screening was to 69 years old. The time horizon was set at 50 years since the estimate survival probability was less than 10% for Chinese women at 85 year of age and the mortality was not available for older age group.

### Key parameter estimates

Stage progression transition probabilities referred to Wong et al. [15]. Age-specific breast cancer incidence was extracted from Chinese Cancer Registry Annual Report 2011 [16]. All cause-mortality was referred to the official 2012 statistical data for China [17]. The sensitivity and specificity for different screen method get from a breast cancer screening study [18] in which 30,935 women enrolled in five cities and 102 breast cancer detected. Key parameter estimates are summarized in Table 1.

Assumption of performance indicators considered as input parameters included stage distribution, attendance rate, and compliance rate for referral for diagnostic tests. The indicators were extracted from the results of a community-based breast cancer screening programs [19].

We calculated quality-adjusted life years (QALYs), which weighted the time spent in each health state by health related quality-of-life weightings from the Hong Kong model [15]. A QALY of 1.0 was defined as “well without breast cancer,” but QALYs of < 1.0 were ranked by cancer stage, with QALYs of 0.95, 0.9, 0.8, 0.7, and 0.3 corresponding to stages DCIS, I, II, III, and IV, respectively.

### Costs

Four major categories were included for the following direct medical costs (Table 1): (1) the cost to community health services; (2) the cost of evaluating abnormal CBE results, including the costs of ultrasound and mammography; (3) the cost of biopsy [20]; and (4) the cost of treating invasive cancer and DCIS (primary treatment) include inpatient cost and outpatient cost. The outpatient cost includes follow-up screening test, radiotherapy, chemotherapy and targeted Therapy and so on in the next year after primary treatment [21, 22].

### Model outcomes

The primary outcome of the model was the incremental cost-utility (ICUR), which was defined as the program’s cost per QALY gained. Model outcomes also included the number of deaths from breast cancer, the number of deaths from other causes, person years of survival adjusted for health quality, and person years of survival with breast cancer (the life years and lifetime costs). The cost-effectiveness threshold: highly cost-effective (less than 1 GDP per capita), cost-effective (1–3 times GDP per capita; 6751 USD -20 272USD), and not cost-effective (more than 3 times GDP per capita) [23]. Future costs and QALYs were discounted at an annual rate of 3% from a societal perspective. The comparator was set as the null scenario of no screening.

### Sensitivity analysis

A Monte Carlo simulation was done with 1000 runs to select values at random from appropriate probabilistic distribution [15] model parameters. Based on the simulation results, cost-effectiveness acceptability curves were presented to the uncertainty in the ICUR caused by variations in parameters such as the attendance, compliance with referral for diagnostic tests, incidence (Please see Additional file 1: Figure S1) and positivity for stage I disease. One-way sensitivity analyses were performed to assess the effects of changes in a given parameter on the model’s outcomes. Expected Value of Partial Perfect Information (EVPPI) was calculated. The acceptability of willingness-to-pay (WTP) triple the annual gross domestic product (i.e., 3 GDPs) per capita per QALY was compared between different cut-off points for the performance indicators.

## Results

### Cost-effectiveness

In the model simulation, annual community-based breast cancer screening from age 35 years to 69 years would avert approximately 177 deaths from breast cancer per 100,000 women compared with no screening (Table 2). In addition, the screening model would detect 1.86 times more DCIS cases would die of other causes compared with the no screening model (e.g., 164 vs. 88 cases per 100,000 women). Compared with no screening, the community-based breast cancer screening would also save approximately 1583 discounted QALYs per 100,000 women screened, equivalent to an average increase in life expectancy of 5.25 years per woman with breast cancer, but for an increase in cost by 10.3 million dollars. Compared with the annual CBE strategy, less frequent screening every two or three years led to small decreases of 4% and 8% in the marginal QALYs, respectively, with corresponding total cost decreases of only 5% and 7%. The ICUR was 8137.29 of every two years screening strategy compare to every three years screening strategy that was higher than the other two ICURs (7075.77 and 8047.96; Table 3).

**Table 2:** Health states and cumulative number for a single simulated cohort of 100,000 Chinese women aged 35 years at the last year of simulation (50th year)

Screen strategy	Well	DCIS	Stage I	StageII	Stage III	Stage IV	Die of BC	BC death avoided (%)	No BC die of other causes	DCIS die of other causes	Invasive BC die of other causes	Screen-detected
No screen	33,068	58	240	184	27	107	2187	–	63,017	88	1024	–
1/1 year	33,097	100	266	172	25	102	2010	8.8	62,986	164	1078	1698
1/2 years	33,097	79	251	173	25	102	2090	4.6	62,987	127	1069	903
1/3 years	33,097	72	246	174	25	102	2118	3.3	62,985	114	1067	580

Abbreviations:*DCIS*; Ductal carcinoma in situ, *BC*; Breast cancer

**Table 3:** The cost-utility analysis of different screening strategy

Screening Strategy	Utility (QALY)	Cost (million USD)	ICUR^a^ (USD/QALY)	ICUR^b^ (USD/QALY)	CU (USD/QALY)
No screen	2388 195	96.08	–	–	40.23
1/3 years	2388 782	100.23	7075.77	7075.77	41.96
1/2 years	2389 034	102.28	7394.60	8137.29	42.81
1/1 year	2389 778	108.27	7701.68	8047.96	45.30

Abbreviations: *CU* Cost/Utility, *ICUR* incremental cost-utility ratio, *QALY* quality-adjusted life-year

ICUR ^a^ based on the no screen strategy; ICUR ^b^ based on the previous screening strategy

### Sensitivity analysis

**Fig. 2:**
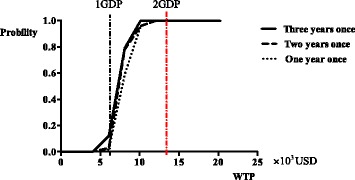
The acceptable curves for different schemes

Figure 2 shows the cost-effectiveness acceptability curves based on different WTP values (the ceiling cost-effectiveness ratio). The probability of the ICUR being below 3 GDPs per QALY saved was 100% for two non-dominated options. The annual screening strategy was also acceptable when the WTP was > 2 GDPs per QALY.EVPPI analysis returned a threshold of zero for most model parameters with a WTP threshold of 3 GDP. This finding suggests that although there is uncertainty around model parameter estimates, reducing this parameter uncertainty is not likely to change the decision about whether routine breast cancer screening should be recommended.

### CEAC for variation in the age-specific cancer incidence

**Fig. 3:**
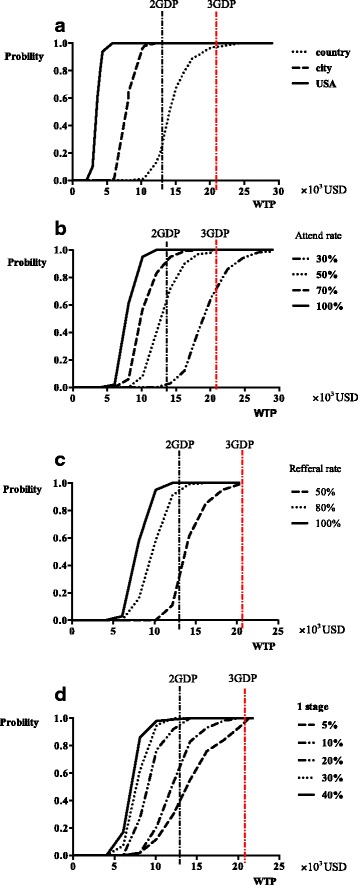
**a** The acceptable curves for different breast cancer incidence. **b** The acceptable curves for different attend-rate. **c** The acceptable curves for referral rate. **d** The acceptable curves regarding to different proportions of stage 1 tumor assumed

When we used U.S. age-specific cancer incidence [24, 25] or age-specific cancer incidence in rural Chinese areas [17] (Table 1) as the input parameters, the cost-effectiveness rankings did not change; however, the probability of it being acceptable was different by area (Fig. 3a). When the incidence of the U.S. population was used in the screening simulation, the probability of the annual screening strategy being cost effective was 100% at a WTP threshold of 1 GDP. But, when the incidence was substituted for urban and rural Chinese areas, the probabilities were 40% and 0%, respectively.

### CEAC for variation in the attendance rate

The attendance rate had a substantial influence on the cost-effectiveness of community-based breast cancer screening. When the value was set to 30%, the probability of the annual screening strategy being cost effective was < 50% at the 3 GDP level of WTP. When the attendance rate was > 50%, the probability for the annual screening strategy being cost effective was > 95% at the 3 GDP level of WTP (Fig. 3b).

### CEAC for variation in the compliance rate for transfer

When compliance with transfer was set at > 50%, the probability for annual screening being cost effective was about 95% at the 3 GDP level of WTP. But, the probability for annual screening strategy being cost effective was > 95% at the 2 GDP level of WTP only when the compliance rate was set at > 80% (Fig. 3c).

### CEAC for variation in detection of earlier stage of tumor

When 10% of stage I tumors were detected on the screening simulation, the probability of the annual screening strategy being cost effective was > 95% for WTP levels > 3 GDPs. When 20% of stage I tumors were detected, however, the probability of the annual screening strategy being cost effective was > 95% at the 2 GDP level of WTP (Fig. 3d).

## Discussion

In this study, we evaluated the effectiveness of an ongoing, community-based, breast cancer prevention program offered by community health services in Tianjin, an urban area in China. The cost-effectiveness analysis showed that the ongoing costs of this screening program were lower than the recommended threshold of triple the GDP per capita per QALY, and that they were cost effective regardless of whether a 1- or 3-year screening interval was used. In addition, it was shown that the annual screening strategy would still be cost effective at the triple the GDP per capita per QALY threshold when the age-specific cancer incidence was as low as that in rural areas. If the anticipated reduction in breast cancer mortality is to be achieved in reality, the following targets will probably need to be met: an attendance rate of at least 50%, compliance with transfer of at least 50%, and an incidence of stage I tumor of at least 10%.

Although the effectiveness of population-based breast cancer screening is of paramount importance, cost-effectiveness analyses are also necessary and play an important role in decision-making for public health policy. A conventional community-based screening strategy for the early diagnosis of breast cancer has been available in Tianjin China since 2005, and there are no data regarding long-term outcomes. The results of the current cost-effectiveness analysis, and the probabilistic approach in particular, show that screening at a one-year interval offers cost-effective when compared with no screening. Indeed, the acceptability curve of the Monte Carlo cost-effectiveness plane suggests that, versus no screening, the probability was 80% for cost effectiveness in the parallel mode given a 2 GDPs per QALY ceiling value with the screening distribution of stage I cancer being 40%. Given that the shortage of human resources in primary care may produce a bottleneck to annual breast cancer screening [26, 27], our data indicate that screening every three years may be a reasonable alternative. In current study comparison only was made between strategies with different frequency and with only CBE as screening test. Strategies with different screening start-age and the other combinations of screen tests were not studied in this model simulation. Age distribution in China was normal with one age peak at 45–49 years, displaying differences from USA and Chinese American with two age peaks [28]. Chinese women present with breast cancer at an earlier age. Kwong et al. reported that 17.6% of the women were younger than 40 years old, and age distribution was significantly different from women in the SEER database [29]. Chinese Anti-Cancer Association recommended the starting age as 40 and the local government sponsored the basic public health service package including CBE for women aged 35–69 years. The additional comparisons show that screening strategies of a later starting age at 40 were more cost-saving compared with that with a younger starting age at 35 and could be more preferable for regions with limited human resources (Please see Additional file 1: Table S1).

It will only be many years after the introduction of a breast screening program that any potential reductions in breast cancer mortality can be expected [30]. In the meantime, it is important that interim outcome measures are monitored to determine whether or not the program is performing satisfactorily. One suitable performance indicator that determines the outcome of a screening program is the attendance rate. The recommended targets for attendance were fulfilled in two previous national breast cancer screening projects in the urban areas of Chengdu (49.0%) and Mianyang (52.1%) in 2008 [31]. However, a cross-sectional study in the same area showed a much lower attendance rate (31.9%). An equally disappointing participation rate (21.7%) was reported by the 2010 China Chronic Disease and Risk Factor Surveillance System, which included data from both urban and rural areas [32]. Comprehensive and prioritized strategies are therefore needed to improve breast cancer screening participation and ensure its cost-effectiveness.

Women with positive CBE results were advised to undergo a combination of USG and MAM for diagnosis, even though these were not covered by the community breast cancer screening package. Compliance with these further investigations also affected the efficacy of the screening strategy. It was identified, that to ensure a probability of 95% for cost effectiveness of the annual screening strategy at the 3 GDP level of WTP, the quality assurance target would need to be 50%. In another breast cancer screening program with a similar design in Qibao Shanghai, about 30% of participants who should have undergone imaging did not [33]. It is important to educate, train, and motivate referring clinicians in community health services of the importance of their role in enabling women to make informed decisions [34, 35].

Finally, CBE was introduced as the initial method of screening for breast cancer in our community-based strategy [36, 37]. Apart from the attendance and transfer compliance rates, the performance of CBE should also be considered relevant to the overall efficacy of the program [36], especially given that measures of screening accuracy are particularly important interim indicators of effectiveness [4]. In our study, we considered the rate of detection of earlier stage tumors to be a suitable indicator of screening efficacy, rather than the overall rate of cancer detection or the screening sensitivity. The annual screening strategy remained cost effective when detection rate of stage I tumor was assumed even lower (10%/20%) and the total proportion of other stages was set at 90%/80% with each proportion for stage II to IV simulated randomly change. Similar screening that relied on initial CBE in Shanghai indicated that a detection proportion of 29% for stage I tumors would be acceptable at a 3 GDP level of WTP [33]. The performance of CBE, however, can only be assured when the involved clinical staff are sufficiently well trained and have appropriate knowledge of the principles of breast cancer diagnosis, management, and screening.

As a major limitation of the current study, it should be noted that any Markov decision model should be validated using external empirical data. However, the screening program still requires long-term follow up to provide this empirical data. To mitigate this, we took care to calibrate the analysis to fit local empirical observations, and most of the parameters assigned to the Markov cycle tree were derived from previous screening program [9]. The Monte Carlo simulation was also done with 1000 runs to select values at random from appropriate probabilistic distributions of model parameters. Three performance indicators for quality assurance were identified in the current study using Monte Carlo simulation, and these might be useful as initial measures of program quality. Moving forward, continuous follow up of the target population is needed over an extended period of time to facilitate a long-term evaluation of its effectiveness [38]. Over-diagnosis was always an important issue when a screening strategy was discussed. However, only over-diagnosis of DCIS was evaluated in current study. The screen-detected breast cancer cases had probability dying of other cause before being clinical occurrences that could be evaluated only when sojourn time was available. Arrospide, A et al. reported that 4% of screen-detected cancers were over-diagnosed [39]. Another study presented that one breast cancer death prevented for about every three over-diagnosed cases identified and treated [40]. When a screening program was discussed, both benefits and harms should be taken into accounted. It should also be noted that the time horizon was set at 50 years in current study and for women elder than 85, the model is not able to predict whether death will be due to BC or other causes that might influence the effectiveness of screening, but it will not cause significant changes. In addition, Markov cohort model was used to compare breast cancer screening strategies with different screening intervals. One important limitation should be noted was Markov cohort model couldn’t track individual patient histories which resulted in the fail to specify the benefit of screening over no screening in individual view. Another useful technique discrete event simulation modeling can track individual patient histories, such that each individual in the economic model can carry a large amount of information which can affect their future treatment options, risk of events and prognosis over time. However, the utilization of discrete event simulation required more data. Due to the lack of age distribution of preclinical phase onset and its mean duration, Markov cohort model was chosen over discrete event simulation. It should be mentioned that Markov cohort model might resulted better estimates for the decision making of the health care resource allocation [41, 42].

## Conclusion

Chinese government and public have high expectations of community health services to protect women’s health by detecting breast cancer early. However, community health services may not always be able to fulfill this role. There is a growing need to develop approaches that reflect the relationships between performance indicators and the feasibility of a screening strategy. Our research suggested that annual community-based breast cancer screening was cost effective for a WTP of 3 GDP based on the incidence of breast cancer in urban city Tianjin, China. Measures are needed to ensure performance indicators to a desirable level for the the cost-effectiveness of breast cancer screening.

## Additional file

Additional file 1: **Figure S1.** Shows the age-specific incidence rate of breast cancer in USA, rural and urban area of China, while **Table S1.** includes the information of cost-utility analysis of different screening strategy among 40–69 years old women. (DOCX 28 kb)

## Abbreviations

CBE: Clinical breast examination
CU: Cost/Utility
DCIS: Ductal carcinoma in situ
GDP: Gross domestic product
ICUR: Incremental cost-utility ratio
MAM: Mammography
QALY: Quality-adjusted life year
USG: Ultrasonography
WTP: Willingness-to-pay
