# Supplementary material for: Quality assurance target for community-based breast cancer screening in China: a model simulation
Source: BMC Cancer. 2018 Mar 7;18:261. doi: 10.1186/s12885-018-4168-1 (PMC5840933; doi:10.1186/s12885-018-4168-1)
Supplement: Supplementary file 1 — Figure S1. Shows the age-specific incidence rate of breast cancer in USA, rural and urban area of China, while Table S1. includes the information of cost-utility analysis of different screening strategy among 40–69 years old women. (DOCX 28 kb) [file 12885_2018_4168_MOESM1_ESM.docx]

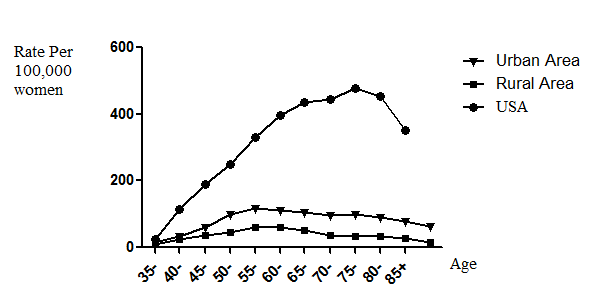


**Figure S1** The age-specific incidence rate of breast cancer in different area

Data sources: USA [1,2]; Rural and urban area of China [3]

Reference

1. DeSantis CE, Fedewa SA, Goding Sauer A, Kramer JL, Smith RA, Jemal A. Breastcancer statistics, 2015: Convergence of incidence rates between black and whitewomen. CA Cancer J Clin. 2016, 66(1):31-42.

2. Surveillance, Epidemiology, and End Results (SEER) Program. SEER public use CD-ROM program. Bethesda, Md: National Cancer Institute, Division of Cancer Prevention and Control, Surveillance Program, Cancer Statistics Branch; 1973–2002.

3. Hao J, Zhao P, Chen WQ. Chinese Cancer Registry Annual Report 2011. 1st ed. Beijing: Military Medical Science Press; 2011.

**Table S1 The Cost-utility Analysis of Different Screening Strategy (screening aged 40-69 years)**

| Start age | Screening Strategy | Utility  (QALY) | Cost  (Million USD) | ICUR ^a^  (USD/QALY) | ICUR^b^  (USD/QALY) | CU  (USD/QALY) |
| --- | --- | --- | --- | --- | --- | --- |
|  | No screen | 2388 195 | 96.08 | - | - | 40.23 |
| 40-69 | 1/3 years | 2388 683 | 99.01 | 6004.10 | 6004.10 | 41.45 |
|  | 1/2 years | 2388920 | 100.82 | 6537.93 | 7637.13 | 42.20 |
|  | 1/1 year | 2389 555 | 105.54 | 6955.88 | 7433.07 | 44.17 |

Abbreviations: CU = Cost/Utility; ICUR = incremental cost-utility ratio; QALY = quality-adjusted life-year

ICUR ^a^ based on the no screen strategy; ICUR ^b^ based on the previous screening strategy
